# The Scenario of Adoption and Foster Care in Relation to the Reproductive Medicine Practice in Asia

**DOI:** 10.3390/ijerph18073466

**Published:** 2021-03-26

**Authors:** Eriko Shiraishi, Seido Takae, Ahmad Mohd Faizal, Kohei Sugimoto, Aikou Okamoto, Nao Suzuki

**Affiliations:** 1 Department of Obstetrics & Gynecology, St. Marianna University School of Medicine, Kanagawa 216-8511, Japan; eri0415s@yahoo.co.jp (E.S.); s2takae@marianna-u.ac.jp (S.T.); drmohdfaizal@ukm.edu.my (A.M.F.); 2 Department of Obstetrics & Gynecology, The Jikei University School of Medicine, Tokyo 105-0003, Japan; aikou7000@gmail.com; 3 Department of Obstetrics & Gynecology, National University of Malaysia (UKM), Kuala Lumpur 56000, Malaysia; 4 Reproduction Center, Dokkyo University Saitama Medical Center, Saitama 343-0816, Japan; ksjog93@gmail.com

**Keywords:** adoption, foster care, in vitro fertilization, surrogacy, gamete donation

## Abstract

In vitro fertilization (IVF) is a hallmark of reproductive medicine. However, the inconclusive outcome leads to marital disharmonies; thus, the choices of gamete donation and surrogacy (GD/S) are often offered. In restricted countries, the child-rearing choice through foster/adoption care is promising, but the uptake remains low. We explore the current reproductive services and adoption scenarios in Asian countries to delineate this issue. The web and literature search using PubMed and Ichushi was conducted in Japanese and English using the keywords “adoption”, “foster care”, “reproductive medicine”, including the interview with the respective Asian clinicians. We found that an established adoption system was seen in China, Malaysia, and the Philippines, mainly due to the restriction of GD/S. Although GD/S were allowed in Thailand, Singapore, and India, the different local affordability of IVF cost led to various adoption system scenarios. Nevertheless, the country’s economic aspect does influence the establishment of adoption care, mainly due to financial support from local government. Otherwise, the significant barrier was the cultural/religious background leading to low adoption rates. We concluded that the adoption option should always be highlighted as an alternative strategy as it synergistically contributes to children’s and infertile couples’ welfare.

## 1. Introduction

Reproductive medicine is a hallmark in the medical fraternity in facilitating medical conception through in vitro fertilization (IVF). Recently, couples have opted for IVF earlier in their married life; thus, not surprisingly, the results were promising. Due to the current adaptation of lifestyles, there is a delay in attempting conception at a more advanced age, ending with a poor outcome [1]. The current scenario leads to a low probability of having their child and poor adherence to continuing the IVF treatment. The Japanese Ministry of Health, Labor, and Welfare recently reported that the average age of couples who received assisted reproductive technology (ART) procedures had the average mean of 40 years old. It is well established that ART therapy’s success beyond 40 years remains low, even with repeated ART cycles [2]. However, these couples tend to continue the ART treatment although anticipating a poor outcome as they are unsure when they should give up [3]. 

As a concern, ART treatment’s goal is mainly pregnancy and childbirth. However, for couples who failed to achieve this, the struggle is real. The overwhelming stress and emotional process often lead to poor quality of life and disharmonies in the marital situation. Therefore, alternative methods such as eggs or sperm donation are often offered, although these options were strictly prohibited in some countries [4]. Moreover, the exit point for them as the “child-rearing” choice is foster care or plenary adoption. Compared to other countries, the level of awareness of adoption in Japan remains low even in the reproductive field due to acceptance and bias [5].

There are various options of “child-rearing” in Japan. Currently, at least 39,047 children live in alternative care in Japan. They were raised independently by social protective care in Japan. The social protective care is broadly divided into two categories; institutional protective care which offers a child care in group facilities, and home protective care based in an individual home [6]. Home protective care includes the adoption system and a foster care system that allows these children to receive personalized home care, although in different terms and conditions. The adoption system’s main measures are to legalize the parent and child status, including formal adoption registration. Once the adoption is finalized, the biological parental right will be dissolved, and the authority of the child belongs to the adopted parent based on the local government act [7]. Hence, this system creates the closest to a biological relationship for both parties. The foster care system also only caters as a temporary shelter for the children. The foster parent will be solely responsible for caring for the child until the biological parent can take over the responsibility [6,7].

Based on the United Nations’ recommendation in the Guidelines for the Alternative Care of Children 2009, it was suggested that when the children cannot return to their biological parents, a permanent family should be sought through adoption. The facilities and foster care are considered alternative situations until a stable family is found [8]. The key to sorting a permanent family for these children is to continue the relationship even after adulthood as permanency care. It is generally considered to have the following order:

1. Biological parents
2. Biological relative or an individual with a remarkably close relationship
3. Domestic adoption
4. International adoption
5. Protective custody of foster care
6. Protective custody by the institution

The numbers 1–4 led to a permanent home for the children. In contrast, the number 5 and 6 policies are adopted to ensure these children receive at least temporary alternative care as quickly as possible [9]. Currently, foster care and adoption systems are still not widely spread and accepted in some Asian countries, including Japan, even among the potential adoption parents like poor IVF outcome couples. Unfortunately, most Asian countries’ culture emphasizes the blood relationship and genetic connection, hence the low uptake of adoption. As a first step, to enhance the foster care and adoption systems’ acceptability in the future, it is necessary to understand the entire situation and solve the remaining unsettled issues [6,9]. There are no reported surveys on the extent of these systems in other Asian countries that have a similar culture to Japan. Therefore, a survey regarding donor gametes, foster care, and adoption in Japan and nearby Asian countries was conducted.

## 2. Materials and Methods

The web searches were conducted in Japanese and English using the keywords “adoption”, “foster care”, “reproductive medicine”, and the name of each Asian country. Literature searches on the web were also done using PubMed and Ichushi (a Japanese medical abstracts search site). We also interviewed and analyzed information obtained from the Asian reproductive medicine clinicians about the adoption and foster care scenarios, including their cultural and religious backgrounds in their respective countries. We anticipate that there is a possibility that the economic status and reproductive medicine practices of each country does impact the adoption and foster care practices. Therefore, each country’s financials, based on per capita GDP [1,8] with a rank of 1–50 as high, 51–100 as middle, and 101–150 as low, were analyzed and tabulated in the table (Table 1).

## 3. Results

The systematic search yielded 28 adoption and foster care works of literature from all included countries based on the keywords used. At least 14 additional web-based information was subsequently added, mainly updating the current adoption laws and regulations in each country. This information was then combined with the verbal interviews from the four respective reproductive medicine clinicians from Thailand, Indonesia, the Philippines, and Malaysia. All this information was consolidated as the main finding.

### Brief Situation of Foster Care and Adoption System in Several Asian Countries 

Japan

In 2015, at least 45,000 infants and young children were eligible for social protective care at that time they were living in institutional protective care, such as group homes for infants or orphanages. However, only 544 finalized plenary adoptions for home protective care were registered in 2015 [6,10]. The majority of couples who engaged with adoption in Japan had failed infertility treatment. Most of them preferred to adopt after they could not achieve pregnancy rather than during the infertility treatment itself even though they knew of the system’s existence earlier. It is justified as most of them would have preferred to have their biological child while pursuing infertility treatment. Previously, Japan’s low adoption rates were related to a lack of knowledge regarding the system. However, the situation is different nowadays, as the infertility couple are well familiar with the adoption process but still reluctant to use the system due to personal preferences [11]. A private organization for adoption is currently formed to facilitate Japan’s adoption system. This is a non-profit organization that works with its fund. They serve as a notification system, provide adoption awareness among potential adoptive parents, and match the potential adoptive parents with their future children [7,11]. However, this service is limited due to financial constraints and human resources shortage. To overcome this, Japanese law introduced a licensing system for this organization in 2018. The license holder will receive financial support from the government to operate, and they need to comply with criteria to be the intermediaries for the adoption system. The license will be renewed accordingly [5,6,7]. Currently, the Ministry of Health, Labor, and Welfare is also trying to provide more information on foster care and adoption to promote home protective care. In improving children’s welfare, the aim is for home protective custody to become the main form of social protective care [5,6,12].

South Korea

Similarly, adoption serves an essential role in social protective care in South Korea. The traditional norm of critical biological children contributed to the limited number of finalized approvals. Interestingly, in history, adoption systems in Japan and South Korea were implemented initially to deal with children who needed shelter following World War II [13]. However, due to the low economic strength, South Korea switched institutional protective care to home protective care at an early stage due to post-war financial difficulties, thus expanding the adoption system’s role compared to institutional protective care funded by the Japanese government [14,15]. In 2013, home protective care accounted for 46% of all social protective care (adoptions 18%, foster care 28%) in South Korea, compared to only 17% of home protective care rate in Japan, and it continues to serve an essential role in social protective care today [14]. Despite that, half of the children needing social protective care are under institutional protective care. Nevertheless, the introduction of “babyboxes” by Joosarang Community Church in 2009 had tremendously increased the number of orphans. It has been reported at least 200 babies per year have been “collected” with an average of 4 babies per week. This scenario leads to an increase in the availability of children’s adoption in South Korea [16]. However, the uptake of adoption remains low, especially with the introduction of special adoption law in 2012, which required the foster parent to register their adoptive children with their name [17,18]. Otherwise, the Confucianism belief also significantly contributes to this scenario as the child’s blood relations are still preferred [18].

China

In China, gametes donation (e.g., sperm and oocytes) are allowed; however, surrogacy is prohibited. Therefore, the adoption option is still relevant in contact with infertility. The legalization of adoption started in the early 1990s, and since that, China has emerged as the most significant source of international child adoption worldwide. However, the trend is decreasing as there is a higher demand for domestic adoption concerning the one-child policy and improvement of China’s economic development [19,20]. In 2017, there were 410,000 orphans in China, of whom 19,000 were adopted. At least 17,000 finalized for domestic adoptions, 103 finalized adoptions were from outside the Chinese mainland (e.g., Hong Kong, Macao, Taiwan), and 2,228 were finalized for international adoption [21]. There was limited evidence regarding adoption issues in the reproductive medicine field. However, it’s postulated that low adoption rates in infertility areas are mainly due to traditional beliefs. Otherwise, most infertility couples in China preferred gamete donation rather than an adoption child [22].

Singapore

Singapore is known as a high achiever in the reproductive field, and most procedures are made available, including gamete donation and surrogacy. However, they face a low number of donors; thus, urge the infertile couples to seek this option outside the country [23]. Therefore, adoption and foster care are still considered relevant among poor prognosis IVF couples. To date, at least 1100 children required social protective care and 430 are currently under the Singapore foster care system. The Singapore government’s policy is to provide home personalized care for these children. Therefore, at least 8 million Singapore dollars (approximately 6 million USD) was allocated for foster care mediation organizations in 2014 to facilitate adoption care [23]. As a result, at least 300 adoptions are finalized each year, and 70% of these children who require social protective care are being brought up in individual homes [24].

Indonesia

Indonesia is the largest Muslim population globally, with approximately 225 million Muslims registered in the national registry [25]. Therefore, the use of donor gametes and surrogates is highly prohibited by law and religion. Nevertheless, due to local policy, women age at and above 40 years old cannot proceed for ART treatment in Indonesia [26]. Consequently, a couple who desire to undergo ART treatment beyond this age limit will often do so in neighboring countries. In 2008, about 288,000 Indonesian couples reported receiving IVF treatment in Malaysia, and another 226,200 of them chose Singapore for IVF treatment. The international IVF treatment considered a higher number of cases than Indonesian local IVF cycle itself as only 1500 Indonesian couples received IVF in Indonesia in 2008 [27]. Like other countries, in Indonesia, adoption is thought to be the last resort should the infertility treatment fail. However, no official adoption system was established in Indonesia as mostly it was widely implemented as religious-based foster care known as “panti-asuhan”. Therefore, there are limited reports made available in this matter [26,27,28].

Philippines

In the Philippines, gamete donation and surrogate birth are made available, but they are strictly controlled by local law and religious belief. In general, the use of donors among infertile couples is almost negligible. Otherwise, the in-vitro fertilization (IVF) cost is expensive, estimated at around USD 5000–10,000. Therefore, most infertility couples cannot afford the IVF, resulting in adoption, which costs only around USD 2000 for a legal fee [29]. Therefore, the adoption system here is active to facilitate the adoption process for potential foster parents with government support. In the latest report, the Philippines managed to finalize at least 7329 adoptions in 2011 [30].

Thailand

In Thailand, gamete donors and surrogates are widely available and well accepted. Therefore, the adoption option is not popular. Children’s adoption is catered for an international couple rather than domestic. Therefore, the adoption report in Thailand was limited [31].

India

India’s reproductive medicine practice is considered the best as most procedures are allowed here, including gamete donation and surrogacy. However, the cost is expensive for the locals; thus, it attracts more of the international crowd [32]. In 2018, India became one of the fertility tourism countries as they offer a cheaper IVF treatment cost (USD 2000–4000), which is almost five times lower than in the western part of the world [33]. Despite that, the adoption system here is well organized. Child adoption was previously considered taboo in India but is now spoken of freely in Indian society. Most of the adoptive parents are recorded from infertile couples. The Central Adoption Resource Authority (CARA) is a part of the Ministry of Women, and Child Care is responsible for facilitating India’s adoption system [33,34]. Adoption eligibility criteria are standard except that the single male is prohibited from adopting a daughter. Furthermore, the adoption law here is integrated with a particular religion; therefore, preferred religious adoption is not allowed (e.g., Muslims, Christians, and Jews). They can act as only guardians under the Guardians and Wards Act, 1890, and are not considered as adopted parents. Therefore, the child can freely choose their pathway and religion once they grow up. Only Indian citizens who are Hindus, Jains, Buddhists, or Sikhs are allowed to adopt a child formally. The adoption is as per the Hindu Adoption and Maintenance Act, 1956, which was enacted as part of the Hindu code bills. In addition to that, the adoption of abandoned, surrendered, or abused children is governed by the Juvenile Justice (Care and Protection of Children) Act, 2015. There is no specific law for international adoption by foreign nationals as it is still governed under Guidelines Governing Adoption of Children 2015. Interestingly, various types of adoption in India depend on communication between the biological and adoptive parent. Open adoption is considered when direct communication ensures both parties’ combined care satisfaction. There is also semi-open adoption, where the communication is done via adoption agency to update the biological parent regarding their children (e.g., picture & letter); and closed adoption where there is no communication between these two parties. In addition to that, a family member’s adoption is considered as intra-family adoption/relative adoption where the adoptive parents are step-parents or from their family members [34].

Malaysia

Malaysia is one Islamic country in Asia with multi-ethnicity and religious belief. Therefore, the government-based reproductive services adhere to Muslim law, thus are prohibited from using donor gametes for fertilization and surrogacy. However, these practices are still possible in a private center for a non-Muslim couple as there is no standardized ART law in Malaysia regarding these matters [35]. The maximum age limit for all the infertility couples for government-funded ART is 40 years old. Otherwise, they can pursue self-funded ART via a private channel with no age limit. Otherwise, in cases of azoospermia (e.g., testicular failure) or poor ovarian insufficiency, commonly due to gonadotoxicity, adoption will be advised [36,37]. The ART center usually will provide a supporting letter to accelerate the adoption process. Adoption in Malaysia is widely practiced and well accepted. The adoption system is handled by the Ministry of Social and Welfare, which caters to suit Islamic law. In Islamic law, the children from Muslim biological parents are prohibited from being adopted by a non-Muslim parent as highlighted under Malaysia Regulation of Adoption Act 1952. In contrast, a Muslim foster parent can adopt non-Muslim children [38]. To date, at least 13,700 children in Malaysia live in the institutions and orphanages. The number is considered small because Malaysia practices “deinstitutionalization” aiming to reintegrate children with their biological families or place them with adoptive parents to personalized home care [39]. Malaysia’s adoption process is cheap as it processes under the adoption registrar with rarely court or law fees being required. The payment for an adoption application processing fee is less than 10 USD. In 2018, at least 7300 adoptions for children were registered in the adoption registry nationwide in Malaysia [40].

## 4. Discussion

In general, our survey regarding the foster care option and adoption in reproductive medicine is challenging. Most of the information was not assessable with insufficient data, and a limited old report was made available. Almost none of the available literature was integrated into the reproductive medicine theme in most Asian countries in our survey. Therefore, additional web searching and interviewing methods were made possible to gather the essential information to make the survey possible. Nevertheless, for some other Asian countries, the information was strictly not accessible and not publicly available; thus, they were excluded. However, we believe that our information can be a precious information source to promote the adoption and foster care system in Asian countries with various cultural backgrounds and economic statuses.

From our survey, the countries with the most home protective care were China, Malaysia, and the Philippines. The common ground for the outcome was the restriction or prohibition of gamete donation and surrogacy in the reproductive medicine practice; thus, their adoption and foster care systems were well established. Compared to Indonesia with similar practices, the survey data were minimal; thus, the adoption care system could not be explored and discussed further. Concerning Thailand and India, they were advanced in reproductive field practice as the implementation of treatment were universal, including gamete donation and surrogacy with different local affordability IVF cost. As the IVF cost is affordable for locals in Thailand, the home protective care ratio tended to be smaller than in other Asian countries. In contrast with India, as the cost of IVF is expensive, the well-accelerated adoption system was implemented to cater to the domestic need. Our survey also postulates that adoption or foster care seemed to have a low priority in the reproductive medicine field due to little awareness among clinicians and uptake among infertility couples [41]. Therefore, it was speculated that the spread of home protective care in Asian countries depended on the trend of reproductive medicine, mainly the accessibility for gamete donation and surrogacy procedure. However, other continents outside Asia were different despite whether gamete donation and surrogacy mothers were made available.

In comparison to the United States of America (USA), although the reproductive environment seemed to be similar to Thailand, foster care and adoption are very well established and accepted with a high number of finalized adoptions recorded every year. The USA government policies of providing adoption awareness at the early beginning of infertility treatment, a tax exemption, and child support for potential adoptive parents were thought to be the major contributors to this scenario [42]. In addition to the USA, we also found the multivariate report on accepting adoption among couples in Nigeria. The analysis concluded that highly educated couples and good household incomes significantly contributed to the higher acceptability of adoption. The analysis also consolidates that a better understanding of the adoption process, coupled with excellent economic resources is deemed essential to raise the adopted children [43]. Nevertheless, our survey remarks that the culture, religion, local law, and the economic background of the Asian countries are vital influencers for the current foster care and adoption system. Moreover, the government sector’s financial support is also crucial to reconstruct the current adoption system and foster care in Asian countries aiming to be as good as other continents regardless of the current reproductive medicine practice.

From the social welfare perspective, these children should be brought up in individual homes rather than institutional-based care to a better quality of life [44]. However, the stability of long-term adoption should be assessed, especially for the adoption mainly in the context of fertility treatment failure. As most infertility couples will adopt children less than five years old, the adjustment is better with a reported adoption breakdown of less than 6% worldwide [45]. Therefore, the adoption option is considered a good alternative with a predictable promising outcome and low breakdown risk. The only pitfall of adopting children at this age is the higher-level involvement of the birth family interest may lead to poor adjustment and higher adoption failure [44,45]. Hence, proper understanding between the biological and adoptive parents is vital to ensure good adoption outcomes for better welfare care. Finally, the awareness and strengthening of the adoption and foster care system are paramount to be blended into reproductive medicine management. There are many potential adoption parents from this area. Ultimate support from all parties, including the government, reproductive medicine clinician, and the infertility couple, is vital to ensure a better outcome for the adoption system in Asian countries.

### Strengths and Limitations of This Study

To our knowledge, this study is the first to consolidate the adoption care scenario with reproductive medicine practice in Asia. Although there are publications of the adoption data, it is not integrated into the value of reproductive medicine practice. Therefore, our study will be a landmark reference for this matter. Our study also has a limitation. The gathering of information was challenging as most Asian countries had recent data regarding adoption/foster care; however, their information from literature was mainly confined to the west scenario rather than Asia, and almost none of the available literature was integrated into the reproductive medicine practice. There was also scanty information to obtain from the web as most of the official government websites were not recently updated. Thus, our data are limited to the scope that we can achieve. Otherwise, most data are based on the non-governmental organization (NGO) website promoting and updating the current issue of adoption/foster care in those countries.

## 5. Conclusions

The adoption and foster care system should always be integrated into the reproductive field management, especially in challenging fertility cases with a guarded prognosis. The choice of adoption should always be highlighted as an alternative option when achieving the pregnancy is deemed impossible, and cessation of the fertility treatment should be offered. The adoption system should never be made accessible to ensure a good quality of life and harmonize a marital situation despite being infertile. Finally, to establish these systems could improve and contribute to the welfare of both children and infertile couples.

## Figures and Tables

**Table 1:** Reproductive medicine practice and social care in the United States and Asian countries.

Country	Oocytes Donation	Sperm Donation	Surrogacy	Economic Status; GDP Per Capita	Population	The Number of Children Who Need Social Care	Foster Care	Adoption	Home Protective Care Ratio to the Number of Children Who Need Social Care	Support for Adopted Families from Government
United States	○	○	○	High	329,892,701 (2019)	430,000 (2016)	200,000 (2016)	69,000 (2016)	77% (2016)	Yes
Japan	△	△	X	High	126,860,301 (2019)	45,000 (2013)	5,190 (2016)	512 (2014)	17% (2013)	No
South Korea	○	○	○	High	51,225,308 (2019)	6834 (2013)	2,265 (2013)	1749 (2013)	60% (2013)	Yes
Singapore	○	○	X	High	5,804,337 (2019)	1100 (2017)	430 (2017)	352 (2014)	46% (2013)	No
Thailand	○	○	○	Middle	69,625,582 (2019)	88,000 (2017)	2535 (2014)	1012 (2017)	44% (2017)	No
China	△	○	X	Middle	1,433,783,686 (2019)	410,000 (2017)	342,000 (2017)	18,820 (2017)	87% (2017)	No
Indonesia	X	X	X	Low	270,625,568 (2019)	1,800,000 (2015)	130,000 (2015)	528 (2009)	65% (2015)	No
The Philippines	△	△	△	Low	108,116,615 (2019)	9446 (2011)	2270 (2011)	7329 (2011)	71% (2011)	No
India	○	○	○	Middle	1,352,642,280 (2018)	56,000,000 (2007)	5682 (2019)	4027 (2019)	6% (2007)	Yes
Malaysia	X	X	X	Middle	31,949,777 (2019)	21,000 (2018)	13700 (2018)	7300 (2018)	84% (2018)	Yes

○ allowable, △ strictly restricted, X prohibited.

## Data Availability

Not applicable.
